# 

**Published:** 1888-07

**Authors:** 


### ANNOUNCEMENTS.

THE AMERICAN OTOLOGICAL SOCIETY will hold its twenty-first annual meeting on the 17th of July, at the Pequot House, New London, Conn.

THE AMERICAN OPHTHALMOLOGICAL SOCIETY will hold its twenty-fourth annual meeting at the Pequot House, New London, July 18 and 19.

THE AMERICAN RHINOLOGICAL ASSOCIATION will hold its sixth annual meeting at Cincinnati, Ohio, on September, 12, 13, and 14.

The fifty-sixth annual meeting of the British Medical Association will be held at Glasgow, on August 7 and three following days. The President-elect is Professor W. T. Gairdner, M. D., LL. D. The Address in Medicine will be delivered by Dr. Clifford Allbutt, of Leeds; the Address in Surgery by Sir G. H. B. Macleod, M. D.; and the Address in Physiology by Dr. J. G. M'Kendrick. An address on "Recent Investigations in Surgery" will be given by Dr. W. Macewen.

Volume LVII.] JULY, 1888. [Number 1.

### The Chicago Medical Journal and Examiner

EDITOR:

S. J. JONES, M.D., LL.D

COLLABORATORS:

CHICAGO:

Professor E. ANDREWS, M.D., LL.D

Professor J. BARTLETT, M.D.

Professor W. T. BELFIELD, M.D.

Professor F. BILLINGS, M.D.

Professor W. H. BYFORD, A.M., M.D.

Professor L. CURTIS, A.M., M.D.

Professor N. S. DAVIS, Jr., A.M., M.D.

Professor O. C. DeWOLF, A.M., M.D

Professor E. C. DUDLEY, A.B., M.D.

Professor C. FENGER, M.D.

Professor J. N. HYDE, A.M., M.D.

Professor E. F. INGALS, A.M., M.D.

Professor F. S. JOHNSON, A.M., M.D.

Professor H. A. JOHNSON, M.D., LL.D.

Professor C. W. PURDY, M.D.

Professor C. G. SMITH, A.M., M.D

Professor E. WING, A.M., M.D

MILWAUKEE, WIS.:

Professor N. SENN, M.D., Ph.D.

WASHINGTON, D. C.:

S. O. RICHEY, M.D.

UNITED STATES ARMY:
SURGEON D. L. HUNTINGTON, M.D.

UNITED STATES NAVY:
SURGEON-GENERAL J. M. BROWNE, M.D.
MEDICAL-DIRECTOR A. L. GIHON, A.M., M.D.

MARINE HOSPITAL SERVICE:
SURGEON S. T. ARMSTRONG, M.D., Ph.D.

